# HER2^Ile655Val^ Single Nucleotide Polymorphism Associated with Early-Onset Breast Cancer Susceptibility: A Systematic Review and Meta-Analysis

**DOI:** 10.31557/APJCP.2021.22.1.11

**Published:** 2021-01

**Authors:** Tung Nguyen Thanh, Bao Song Nguyen Tran, Ai Phuong Hoang Thi, Thang Tran Binh, Thong Ba Nguyen, Tam Le Minh, Quoc Huy Nguyen Vu, Thuan Dang Cong

**Affiliations:** 1 *Institute of Biomedicine, Hue University of Medicine and Pharmacy, Hue University, 6 Ngo Quyen Street, Hue, Vietnam. *; 2 *Faculty of Basic Science, Hue University of Medicine and Pharmacy, Hue University, 6 Ngo Quyen Street, Hue, Vietnam. *; 3 *Department of Histology, Embryology, Pathology and Forensic, Hue University of Medicine and Pharmacy, Hue University, 6 Ngo Quyen Street, Hue, Vietnam. *; 4 *Faculty of Public Health, Hue University of Medicince and Pharmacy, Hue university, 6 Ngo Quyen Street, Hue, Vietnam. *; 5 *Department of Medical Bioscience, Soonchunhyang University Hospital Bucheon, Bucheon 14584, Republic of Korea. *; 6 *Department of Obstetrics and Gynecology, Hue University of Medicine and Pharmacy, Hue University, 6 Ngo Quyen Street, Hue, Vietnam. *

**Keywords:** Breast cancer, early, onset, HER2^Ile655Val^, meta-analysis, single nucleotide polymorphism

## Abstract

**Background:**

Human epidermal growth factor receptor 2 (HER2) plays an important role in the development and progression of breast cancer. To understand the precise association, this meta-analysis was conducted to estimate the association between HER2^Ile655Val^ single nucleotide polymorphism (SNP) and susceptibility to early-onset breast cancer.

**Methods:**

A comprehensive database retrieval from PubMed, Embase, Web of Science and Google Scholar was pooled to investigate links between the HER2^Ile655Val^ SNP and risk of breast cancer. Adjusted odds ratios (ORs) with 95% confidence intervals (CIs) were estimated to appraise the association under the additive model (Ile vs. Val), dominant model (Val/Val + Ile/Val vs. Ile/Ile), and recessive model (Val/Val vs. Ile/Val + Ile/Ile).

**Results:**

Seventeen relevant studies with 11,749 cases and 8,105 controls were finally included. We found that HER2^Ile655Val^ SNP is associated with an increased risk of breast cancer in an additive and dominant model. In the subgroup analysis with age stratification, a significant association between the HER2 codon 655 SNP and the risk of breast cancer was found in young women in an additive, dominant, and recessive model; conversely, no significant associations were indicated in older women. In the breast cancer subgroup, HER2^Ile655Val^ SNP was significantly associated with younger age women with breast cancer in the dominant model. In contrast, no association between the HER2 codon 655 SNP and age was found in control populations.

**Conclusion:**

Our findings suggest that the Val allele in HER2 codon 655 SNP is strongly associated with breast cancer susceptibility in the young female population and is also significantly associated with younger age in women with breast cancer. HER2^Ile655Val^ SNP might be a susceptibility factor that favours early-onset breast cancer.

## Introduction

Breast cancer is the most common cancer among women, with an increasing incidence in most countries, representing a public health threat (Momenimovahed and Salehiniya, 2019; Zahmatkesh et al., 2016). In the United States, breast cancer caused 42,000 deaths in 2017 (Siegel et al., 2020). Clearly, there is a link with ageing, especially among women aged 45 to 65, and it is increasing among younger women (Bouchardy et al., 2007; Dobi et al., 2011; Zubor et al., 2006). Breast cancer under the age of 40 accounts accounts for 3-7% of all breast cancer cases in women (Anders et al., 2009; Loman et al., 2001). In a recent analysis, breast cancer patients aged under 30 and 30-39 years were found to have a significantly lower survival rate than those aged 40-49 and 50-59 (Chen Hai-long et al., 2016; Paluch-Shimon et al., 2020).

Human epidermal growth factor receptor 2 (HER2), also known as c-erbB2 and neu, is located on human chromosome 17q21 and is responsible for encoding a 185-kDa cross-membrane glycoprotein receptor. HER2 belongs to the ErbB family of growth factor receptors with intrinsic tyrosine kinase activity. The members of this family take the form of homodimer and heterodimer when activated via cell growth, specifically chemical and invasion (Friedlander et al., 2009; Yarden and Sliwkowski, 2001). HER2 overexpression is seen in breast cancer, gastric cancer, and ovarian cancer (Tai et al., 2010). HER2 targeted therapies have significantly enhanced the clinical outcome for HER2-positive breast cancer patients (Incorvati et al., 2013; Sidaway, 2020). Targets in downstream or resistant pathways of particular interest in HER2-positive breast cancer include mTOR, PI3K, IGF-1R, Akt, HSP90, and VEGF that allow cell development, survival, and differentiation (Agus et al., 2005; Arteaga et al., 2012; Franklin et al., 2004; Nahta et al., 2006). 

Single nucleotide polymorphisms (SNPs) residing in regulatory or functionally relevant gene regions may affect protein function (Frank et al., 2005). HER2^Ile655Val^ SNP has been identified in the transmembrane domain-coding region of the *HER2* gene at codon 655, encoding either isoleucine (Ile: ATC) or valine (Val: GTC) (Krishna B Madhu et al., 2018; Ozturk et al., 2013). Substitution of these two amino acids can alter the hydrophobicity of proteins, affecting the shape stability of the regions in the protein (Ameyaw et al., 2002; Papewalis et al., 1991). Fleishman et al. found that substitution of Val for Ile in this position of the transmembrane region will destabilize the formation of active HER2 dimers, leading to reduced receptor activation and tyrosine kinase activity, even under conditions of HER2 overexpression (Fleishman, 2002). The association between the HER2^Ile655Val^ SNP and the risk of breast cancer has been widely investigated in populations worldwide (Al-Janabi et al., 2015; de Almeida et al., 2018; Parvin et al., 2017; Watrowski et al., 2015). 

The association between HER2^Ile655Val ^SNP and the risk of early-onset breast cancer has also been investigated; however, these results are inconclusive and controversial. Several articles have shown the association of HER2^Ile655Val^ SNP with an increased risk of early-onset breast cancer in Chinese, Australian, and Taiwanese women (Lee Su-Chen et al. 2008; Montgomery et al. 2003; Naidu Rakesh et al., 2008; Xie et al., 2000). Nonetheless, the association has not been observed in other studies (An et al., 2005; Kara Nurten et al., 2010; Zubor et al., 2006). In the present meta-analysis, we aimed to obtain a more reliable estimate of the association between HER2^Ile655Val ^SNP and susceptibility to early-onset breast cancer.

## Materials and Methods

*Search strategy and identification of relevant studies*

We systematically searched electronic databases including PubMed, Embase, Web of Science, and Google Scholar to identify all relevant articles with either single or combined search terms: “human epidermal growth factor receptor 2”, “Her2”, “ERBB2”, “neu”, “EGFP2”, “single nucleotide polymorphism”, “SNP”, “polymorphism”, “Ile655Val”, “rs1136201”, “Herceptin receptor polymorphism” and “breast cancer”. Records were included up to 1 January 2020 and screened based on title and abstract with duplicates removed. Full-text papers were independently reviewed and selected to identify the relevant articles for the meta-analysis by three of the authors (T.N.T, B.S.N.T, and A.P.H.T).

*Criteria for inclusion and exclusion*

Studies that fulfilled the inclusion criteria were considered eligible for this meta-analysis. Studies were selected when they: (a) evaluated the association between HER2^Ile655Val ^SNP and breast cancer risk; (b) included allelic or genotype frequencies; (c) provided results with age stratification; (d) represented original data; (e) used a cohort study design, and (f) were written in English. Papers were excluded when they: (a) described a review, case report or conference abstract; (b) did not contain original data or were irrelevant to the current analysis; (c) did not provide genotypic distribution or allele frequency data.

*Data Extraction*

The extracted data elements included the year of publication, ethnicity, number of cases and control samples, cut-off age, genotype distribution, allele frequency for each case, control, and age stratification groups. The genotype distribution of HER2^Ile655Val^ was assessed using Hardy-Weinberg equilibrium. 

*Statistical analysis*

The meta-analysis was performed using Stata SE version 13.1 software (StataCorp, College Station, TX, USA). Random-effects models were used to synthesise the association between the HER2^Ile655Val^ SNP and susceptibility to breast cancer. The heterogeneity study was described using the Higgins I^2^ metric. An I^2^ value of 0% was considered to mean no observed heterogeneity, while a value greater than 50% was considered substantial heterogeneity.

The strength of association was assessed by calculating adjusted odds ratios (ORs) with 95% confidence intervals (CIs). Pooled ORs were performed for the allele genetic (additive) model (allele Val vs Ile), dominant model (Val/Val + Val/Ile vs Ile/Ile), and recessive model (Val/Val vs Val/Ile + Ile/Ile), respectively. The results of all included studies were summarised using a conventional forest plot. Publication bias was estimated using funnel plot asymmetry and Egger’s linear regression test. A P-value < 0.05 was considered as representative of statistical significance.

*Subgroup analysis*

We employed subgroup analysis by age stratification (young and advanced age) and by study participants (breast cancer patients and control population). To investigate the association between HER2^Ile655Val^ SNP and breast cancer susceptibility in young and advanced age populations, subgroup analysis was performed by calculating adjusted ORs comparing cases and controls. To investigate the association between HER2 codon 655 polymorphism and the age of onset, subgroup analysis was performed by calculating adjusted ORs comparing older and younger participants in each breast cancer patient and control group.

## Results

*Characterisation of eligible studies*

**Figure 1:**
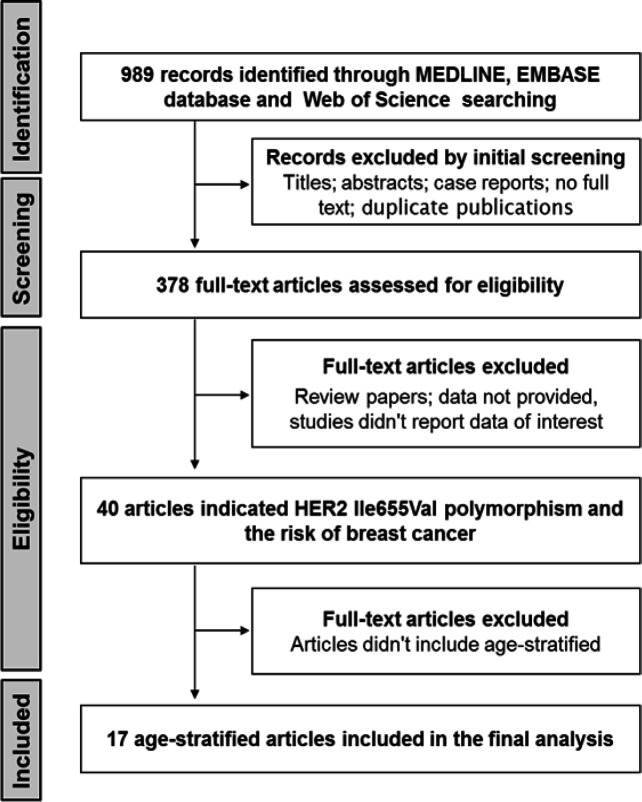
The Selection Process of Papers for the Meta-Analysis Study

The initial search identified 989 potential records from electronic databases, and 378 full-text articles were assessed for eligibility. Of these, 40 articles addressed HER2^Ile655Val ^SNP and the risk of breast cancer. Finally, 17 observational studies that met the inclusion criteria were selected for the meta-analysis (Frank et al., 2005; Han et al., 2014; Kara et al., 2010; Lee et al., 2008; Mutluhan et al., 2008; Naidu et al., 2008; Nelson et al., 2005; Ozturk et al., 2013; Papadopoulou et al., 2007; Pinto et al., 2004; Qu et al., 2008; Tommasi et al., 2007; Wang-Gohrke and Chang-Claude, 2001; Watrowski et al., 2015; Xie et al., 2000; Zubor et al., 2008; Zubor et al., 2006). The detailed steps in the literature selection process are shown in Figure 1.

The general characteristics of the studies in the present analysis are shown in Table 1. We identified a total of 17 age-stratified studies, which included 11,749 breast cancer cases and 8,115 controls, had a cut-off age from 40 to 55 (46.53 ± 3.84), and were conducted over a period of 15 years from 2000 to 2015. The distribution of the HER2^Ile655Val^ genotype in the control groups from the studies was consistent with Hardy-Weinberg equilibrium.

*Association between HER2* ^Ile655Val^ *SNP and breast cancer susceptibility in worldwide populations*

**Figure 2:**
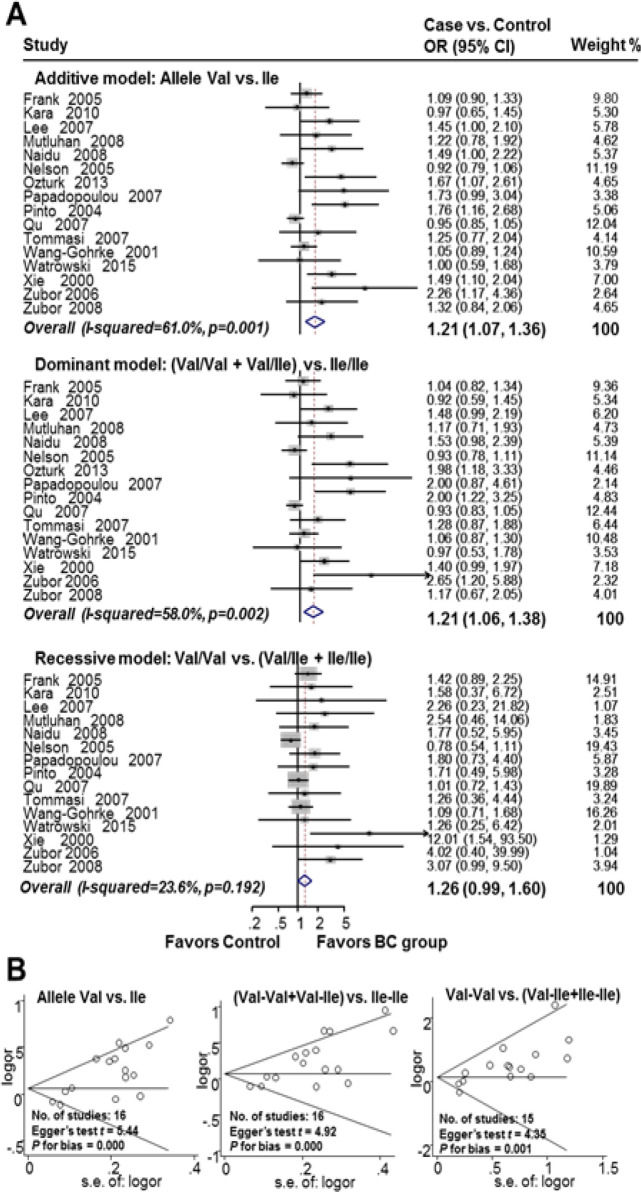
Association between HER2^Ile655Val ^SNP and Breast Cancer Risk Worldwide. A. Forest plot for the association between HER2^Ile655Val^ SNP and breast cancer risk; B. Funnel plot evaluating publication bias among studies included in the meta-analysis

The association between HER2^Ile655Val^ SNP and the risk of breast cancer in worldwide populations is shown in Figure 2. The meta-analysis showed that the HER2 codon 655 Val allele was significantly associated with an increased risk of breast cancer in an allele genetic model (additive model, OR 1.21, 95% CI 1.07-1.36; I^2^=61.0%; n=16). There was a 21% significant increase in the risk of breast cancer in subjects who were Val carriers (Ile/Val and Val/Val) (dominant model, OR 1.21, 95% CI 1.06-1.38; I^2^=58.0%; n=16). The recessive model HER2 codon 655 was not associated with the risk of breast cancer (OR 1.26, 95% CI 0.99-1.60; I^2^=23.6%; n=15). There was publication bias in the studies (Begg’s funnel plot was symmetric; additive model, Egger’s test t=5.44, P for bias=0.000, n=16; dominant model, Egger’s test t=4.92, P for bias=0.000, n=16; recessive model, Egger’s test t=4.35, P for bias=0.001, n=15).

*HER2* ^Ile655Val^ *SNP is associated with increased breast cancer susceptibility in young women, but not in older women*

**Figure 3:**
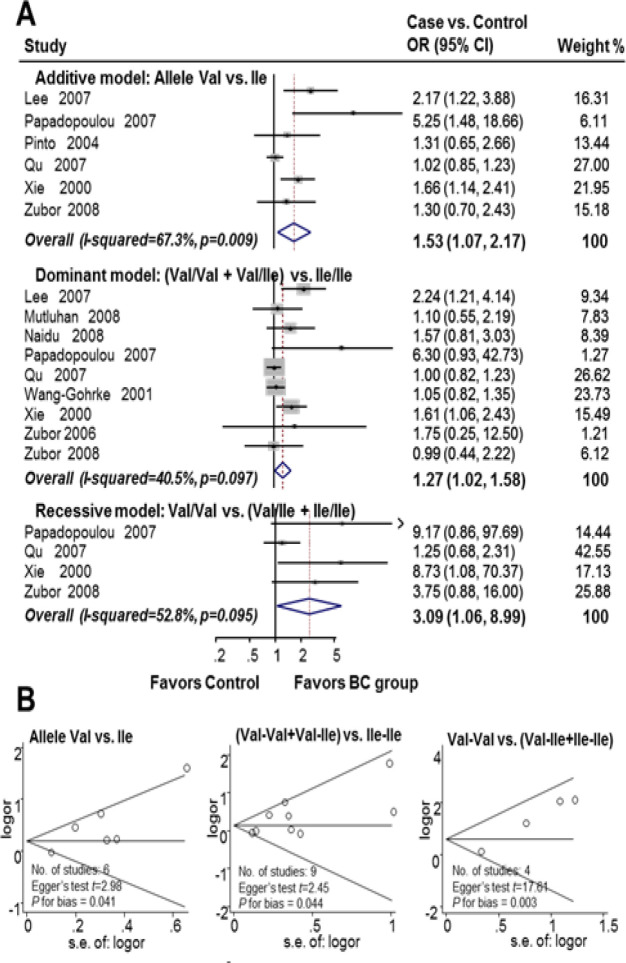
HER2^Ile655Val^ SNP is Associated with an Increased Breast Cancer Risk in Young Women. A. Forest plot for the association between HER2Ile655Val SNP and breast cancer risk in young women; B. Funnel plot evaluating for publication bias among studies included in the meta-analysis

In the subgroup meta-analysis by age stratification, HER2 codon 655 polymorphism was significantly associated with breast cancer in young women under all genetic models, including the additive model (OR 1.53, 95% CI 1.07-2.17; I^2^=67.3%; n=6), dominant model (OR 1.27, 95% CI 1.02-1.58; I^2^=40.5%; n=9), and recessive model (OR 3.09, 95% CI 1.06-8.99; I^2^=52.8%; n=4). There was a significant 27% increase in the risk of breast cancer in young female Val carriers (Ile/Val and Val/Val). Publication bias was found in the studies (Begg’s funnel plot was symmetric; additive model, Egger’s test t=2.98, P for bias=0.041, n=6; dominant model, Egger’s test t=2.45, P for bias=0.044, n=9; recessive model, Egger’s test t=17.61, P for bias=0.003, n=4) (Figure 3). In contrast, there was no statistically significant association between HER2^Ile655Val^ SNP and breast cancer susceptibility in the subgroup of older women (additive model, OR 1.21, 95% CI 0.89-1.66, I^2^=60.9%; n=6; dominant model, OR 1.07, 95% CI 0.90-1.27, I^2^=17.8%; n=9; recessive model, OR 0.95, 95% CI 0.65-1.39, I^2^=0.0%; n=4) (Supplementary Figure 1).

*HER2* ^Ile655Val^ *SNP is associated with increased early-onset breast cancer susceptibility*

**Figure 4:**
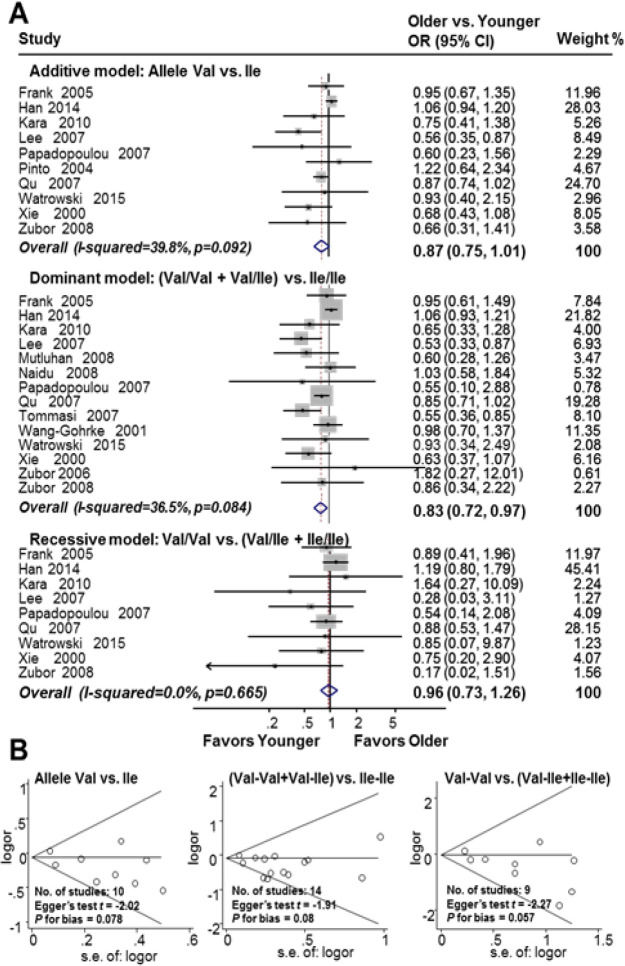
HER2^Ile655Val^ SNP is Associated with an InCreased Risk of Early-Onset Breast Cancer. A. Forest plot for the association between HER2Ile655Val SNP and an increased risk of early-onset breast cancer; B. Funnel plot evaluating for publication bias among studies included in the meta-analysis

Subgroup meta-analysis by study participants was utilised to investigate the association between HER2^Ile655Val^ SNP and the age at onset of breast cancer. Figure 4 summarises the results from the subgroup meta-analysis, calculating adjusted ORs comparing older and younger participants in the breast cancer population. There was a significant 17% increase in the risk of early onset of breast cancer in patients who were Val carriers (Ile/Val and Val/Val) (dominant model, OR 0.83, 95% CI 0.72 to 0.97; I2=36.5%; n=14). Meanwhile, no significant association of HER2^Ile655Val^ SNP polymorphism with the age of onset was found in the subgroup of breast cancer women under an additive model (additive, OR 0.87, 95% CI 0.75-1.01, I^2^=39.8%; n=10) and a recessive model (OR 0.96, 95% CI 0.73 to 1.26, I^2^=0.0%; n=9). There was no publication bias in the studies (Begg’s funnel plot was symmetric; additive model, Egger’s test t=-2.02, P for bias=0.078, n=10; dominant model, Egger’s test t=-1.91, P for bias=0.08, n=14; recessive model, Egger’s test t=-2.27, P for bias=0.057, n=9). Furthermore, subgroup meta-analysis of the control population indicated no significant association between HER2^Ile655Val ^SNP and the age of participants was found in control women under all genetic models (additive, OR 0.94, 95% CI 0.75 to 1.18; I2=28.1%; n=6; dominant, OR 0.94, 95% CI 0.83 to 1.07; I2=0.0%; n=9, and recessive, OR 1.19, 95% CI 0.70 to 2.02; I^2^=2.0%; n=3) (Supplementary Figure 2).

**Table 1:** The Characteristics and Distribution of Studies Examining the Association between HER2^Ile655Val^ Polymorphism and the Risk of Early-Onset Breast Cancer

No.	Study ID	Ethnic group	Cut off age	n		Genotype Distribution						Allele Distribution			
				Case	Control	Case			Control			Case		Control	
						Ile/ Ile	Ile/ Val	Val/ Val	Ile/ Ile	Ile/ Val	Val/ Val	Ile	Val	Ile	Val
1	Frank 2005	German	40	347	960	186	132	29	525	377	58	504	190	1427	493
2	Han 2014	Chineese	50	4167		2984	1086	97				7054	1280		
3	Kara 2010	Turkish	50	204	192	153	46	5	141	48	3	352	56	330	54
4	Lee 2007	Taiwanese	45	424	318	341	80	3	273	44	1	762	86	590	46
5	Mutluhan 2008	Turkish	50	166	208	128	34	4	166	40	2	290	42	372	44
6	Naidu 2008	Malaysian	50	230	200	165	57	8	159	37	4	387	73	355	45
7	Nelson 2005	Europian	55	1094	976	637	396	61	551	356	69	1670	518	1458	494
8	Ozturk 2013	Turkish	40	118	128	61	57	0	87	41	0	179	57	215	41
9	Papadopoulou 2007	Greeks	45	56	45	15	22	19	19	16	10	52	60	54	36
10	Pinto 2004	Portuguese	46	152	146	88	57	7	107	35	4	233	71	249	43
11	Qu 2007	Chineese	45	3012	3004	2298	648	66	2252	687	65	5244	780	5191	817
12	Tommasi 2007	Caucasian	45	628	169	433	181	14	125	41	3	523	104	145	23
13	Wang-Gohrke 2001	Caucasian	45	615	1078	360	219	36	646	374	58	939	291	1666	490
14	Watrowski 2015	Austrian	50	80	100	51	26	3	63	34	3	128	32	160	40
15	Xie 2000	Chineese	45	339	359	243	85	11	280	78	1	571	107	638	80
16	Zubor 2006	Slovakian	45	47	60	22	22	3	42	17	1	66	28	101	19
17	Zubor 2008	Caucasian	45	70	172	38	25	7	100	66	6	101	39	266	78

## Discussion

Several studies have independently discovered the association between HER2^Ile655Val ^SNP and different types of benign and malignant tumours, including breast cancer, prostate cancer, colorectal cancer, osteosarcoma, gastric cancer, uterine cervical carcinoma, and fibroadenoma (Chen et al., 2014; Kruszyna et al., 2010; Kuraoka et al., 2003; Liang et al., 2009; Ozturk et al., 2013; Xin et al., 2015; Yokomizo et al., 2005; Zubor and Karol Kajob, 2008). Riaz et al., (2016) conducted a meta-analysis research and concluded that HER2^Ile655Val^ SNP is significantly correlated with six major types of cancer (breast, ovary, uterine, lung, thyroid, and gastric), suggesting that carriers of the Val allele and Val/Val genotype may be linked with an elevated risk of these cancers. This meta-analysis found that HER2^Ile655Val^ SNP is associated significantly with an increased risk of breast cancer, particularly in young women. Our findings are consistent with results from previous meta-analyses by Tao et al., (2009), Lu et al., (2010), Wang et al., (2013), Chen et al., (2014), and Krishna et al., (2018). However, a meta-analysis by Ma et al., (2011) revealed that HER2^Ile655Val^ SNP is not associated with breast cancer susceptibility. Our results further demonstrate the potential contribution of this single nucleotide polymorphism to the oncogenesis of breast cancer. 

Prior studies independently found a correlation between the high presence of the Val allele in the codon 655 of the *HER2* gene and the onset of breast cancer (Joni et al., 2003; Karen et al., 2003; Papadopoulou, 2007; Xie et al., 2000). Additionally, Millikan et al., (2003) and Tommasi et al., (2007) reported that there was a strong association between the variant allele 655Val and breast cancer in younger women when combined with family history (Robert and Dressler, 2003; Tommasi and Vita Fedelea, 2007). However, other published data reveal the opposite results, with no significant correlation between the HER2^Ile655Val^ genotype and the risk of early-onset breast cancer in patients 40 to 50 years (An Hee Jung et al., 2005; Baxter and Campbell, 2001; Chan et al., 2002; Nurten Kara, 2010; Watrowski et al., 2015). We conducted the present meta-analysis, which collected data from 17 age-stratified articles with a cut-off age value from 40 to 55 years old (46.53 ± 3.84). We found that in young women, HER2 codon 655 polymorphism was strongly and significantly associated with breast cancer in all genetic models (additive, dominant, and recessive), which was in contrast to the results from a subgroup of older women. In the breast cancer population, the dominant model of HER2^Ile655Val^ SNP was shown to be significantly associated with younger age. Therefore, the high presence of the Val allele in codon 655 of the *HER2* gene might be an explanation for the increasing frequency of younger age onset of breast cancer. 

The molecular mechanism of HER2^Ile655Val^ SNP, a non-polar to non-polar amino acid mutation, has been investigated in previous studies. Using computational exploration, Fleishman et al., (2002) proposed that the transmembrane region of the HER2 homodimer can exist in two stable conformations, either in an active or inactive form. The dimer mediated by the C-terminal dimerisation motif is more stable than the dimer formed by the N-terminal motif. The authors found that substitution of Val for Ile in this position of the transmembrane region will destabilise the formation of active HER2 dimers mediated by the N-terminal dimerisation motif and lead to reduced receptor activation and tyrosine kinase activity. However, the presence of the Val allele could reinforce the stabilisation of the receptor’s active state, which results in augmentation of autophosphorylation, hyper-active tyrosine kinase, and cellular proliferation (Fleishman, 2002). Bocharov and colleagues researched the spatial structure of the dimeric transmembrane domain of the HER2 protein. They found that the Ile655Val variant can excessively stabilise the ErbB2 active dimeric state due to substitution of the bulk side chain of Ile with the smaller Val, thus allowing tighter TM helix packing (Bocharov et al., 2008). In another experiment, Tanaka et al. assessed the role of amino acid substitutions in the conformational stability of human lysozyme protein via thermodynamic analysis at high temperature and very low pH. They showed that in constructed isoleucine to valine mutants, the stability of mutant proteins was reduced compared to that of the wild-type protein (Takano, 1995).

Molecular mechanisms of the early onset state have been studied in certain types of cancer. Several candidate genes and signalling pathways have been found in the early onset of colorectal cancer. *REG1A, CK20*, and *MAP3K8* gene expression was shown to be related to early-onset colorectal cancer formation (Tezcan et al., 2016). Using PPI network analysis, Zhao et al., (2019) suggested that early-onset colorectal cancer is associated with vascular smooth muscle contraction signalling pathway. They also identified seven hub genes, namely, *ACTA2, ACTG2, MYH11, CALD1, MYL9, TPM2*, and *LMOD1*, along with this signalling pathway. Recently, using weighted gene co-expression network analysis and other analysis methods, Mo et al. identified seven genes (*SPARC, DCN, FBN1, WWTR1, TAGLN, DDX28* and *CSDC2*) associated with the development and prognosis of early-onset colorectal cancer. These genes may serve as novel biomarkers for the diagnosis of early-onset colorectal cancer (Mo et al., 2020). In prostate cancer, a study conducted by Weischenfeldt et al. found the genomic alteration landscapes of early-onset prostate cancer compared to older-onset cancer. They discovered that early-onset prostate cancer possesses a higher frequency of balanced structural rearrangements, with a specific abundance of androgen-regulated *ETS* gene fusions and concluded that *ETS* fusion genes are signs of early-onset prostate cancer (Weischenfeldt et al., 2013). Furthermore, Gerhauser et al., (2018) demonstrated the role of androgen receptor-driven rearrangements, an early APOBEC-driven mutational mechanism, and *ESRP1* gene duplication that contributed to the pathogenesis seen in early-onset prostate cancer. Nevertheless, the molecular mechanism involved in the pathogenesis of early-onset breast cancer is to date poorly understood and needs to be studied further.

In conclusion, our analyses showed an association between HER2^Ile655Val^ SNP and an increased risk of breast cancer. In addition, we propose that HER2^Ile655Val^ SNP might be considered as a susceptibility factor for early-onset breast cancer. Further molecular studies are required to reveal the mechanism of this correlation. 
